# Autoantibodies against glucose-regulated protein 78 as serological diagnostic biomarkers in hepatocellular carcinoma

**DOI:** 10.3892/ijo.2012.1515

**Published:** 2012-06-12

**Authors:** QING SHAO, PENGFEI REN, YANG LI, BO PENG, LIPING DAI, NINGJING LEI, WU YAO, GANG ZHAO, LINGGEN LI, JIANYING ZHANG

**Affiliations:** 1 Department of Biological Sciences, The University of Texas at El Paso, El Paso, TX 79968, USA;; 2 The First Affiliated Hospital of Heilongjiang University of Chinese Medicine, Harbin, Heilongjiang 150040;; 3 Henan Key Laboratory of Tumor Epidemiology and College of Public Health, Zhengzhou University, Zhengzhou, Henan 450052, P.R. China

**Keywords:** autoantibody, GRP78, hepatocellular carcinoma, biomarker, immunodiagnosis

## Abstract

Hepatocellular carcinoma (HCC) is a type of cancer with a very poor prognosis. Although α-fetoprotein (AFP) is the most effective marker available to detect HCC, the sensitivity and specificity are not optimal. Therefore, there is a need for the development of more sensitive and specific methods that can supplement AFP in the early detection of this cancer. In this study, autoantibody responses to glucose-regulated protein 78 (GRP78) were evaluated by enzyme-linked immunosorbent assay (ELISA), western blotting and indirect immunofluorescence assay in sera from patients with HCC, liver cirrhosis (LC) and chronic hepatitis (CH), as well as from normal human individuals. Immunohistochemistry (IHC) with tissue array slides was also preformed to analyze protein expression profiles of GRP78 in HCC and control tissues. The prevalence of autoantibodies against GRP78 was 35.5% (27/76) in HCC, which was significantly higher than that in LC, CH and normal human sera (NHS; P<0.01). The average titer of autoantibodies against GRP78 in HCC sera was higher compared to that in LC, CH and NHS(P<0.01). When both autoantibodies against GRP78 and AFP were used simultaneously as diagnostic markers, sensitivity reached 71.4%. Our data indicate that anti-GRP78 autoantibodies may be potential diagnostic markers for HCC, especially in conjunction with AFP.

## Introduction

Hepatocellular carcinoma (HCC) is the fifth most common tumor worldwide and the third most common cause of mortality from tumor, causing approximately 600,000 cases of mortality worldwide each year (1). The high mortality rate of HCC can in part be attributed to a lack of diagnostic methods that allow for early detection. It is well known that early detection and treatment can improve the survival rate of patients with HCC (2). Several studies have demonstrated that surveillance for high risk individuals, such as chronic hepatitis (CH) and liver cirrhosis (LC) patients, is a vital method to detect HCC earlier and to provide optimal opportunity for treatment (3), which has been shown to improve patient survival (4–6). Although α-fetoprotein (AFP) is the most effective serological marker available to detect HCC, its sensitivity and specificity are not optimal (7). Therefore, it is imperative to develop more effective methods, especially at the early stage, for the diagnosis of HCC.

Previous studies have shown that in the case of HCC, antecedent LC and CH are common precursor conditions and during transition to malignancy some patients develop autoantibodies that were not present during the preceding chronic liver disease phase (8). These autoantibodies to tumor-associated antigens (TAAs), which are known as ‘reporters’ from the immune system, identify the antigenic changes in cellular proteins involved in the transformation process (9). Serological screening of autoantibodies to TAAs may be used as an effective method to identify patients with HCC at an early stage (10). With the widespread application of the technologies, more TAAs have been identified in HCC, and also anti-TAA autoantibodies have been detected in sera from patients with HCC. The concern is that the sensitivity and specificity of autoantibodies to single TAA as a diagnostic marker in HCC are currently still low and insufficient for the diagnosis of HCC (10). However, using a mini-array of multiple TAAs to detect autoantibodies simultaneously may enhance the sensitivity and specificity, which may be a potential valuable approach for cancer diagnosis (11). Previous studies in our lab have demonstrated that the final cumulative prevalence of autoantibodies to TAAs can reach 66.2% by using an array of 10 TAAs including c-myc, p53, cyclin B1, p62, Koc, IMP-1, survivin, p16, Sui1 and RalA, to detect autoantibodies in sera from patients with HCC (12). In order to improve both the sensitivity and specificity of anti-TAA autoantibodies as biomarkers in HCC detection, the major task is to continue identifying and validating more valuable TAAs in HCC to add in the mini-array of TAAs which we have created in previous studies, for optimizing the combination of the mini-array of TAAs in HCC.

Glucose-regulated protein 78 (GRP78), also referred to as immunoglobulin heavy chain binding protein (BiP), is a chaperone protein belonging to the HSP70 protein family, which resides primarily in the lumen of endoplasmic reticulum (ER) (13,14). GRP78 is a vital functional protein in the physiological and pathological conditions of ER, which can facilitate protein folding, assembly, transport, calcium homeostasis, and can also regulate ER stress signaling under the ER stress (14,15). Overexpression of GRP78 in certain types of tumors, such as lung, breast, stomach, prostate and HCC, has been widely reported (16–20). Many studies have indicated that the function of GRP78 is closely related to tumor proliferation, survival, metastasis, apoptosis, angiogenesis, and chemoresistance (18,21–27). Importantly, ectopic expression of GRP78 on the cancer cell surface, but not in normal cells, has been revealed, suggesting that GRP78 may be a potential target of cancer therapy (28–30). Furthermore, autoantibodies against GRP78 have been detected at high levels in the sera from patients with prostate and gastric cancers (31–33). Whether autoantibodies against GRP78 can also be detected in sera from patients with HCC and whether autoantibodies against GRP78 can be used as a serological diagnostic markers in HCC remain to be investigated. This study determines the prevalence of anti-GRP78 autoantibodies in sera from patients with HCC, LC and CH, as well as from normal human sera (NHS), to further validate the diagnostic value of anti-GRP78 autoantibodies in the immunodiagnosis of HCC.

## Materials and methods

### Sera and general information

All sera used in this study, 76 sera of HCC, 30 sera of LC, 30 sera of CH, and 86 NHS, were obtained from the serum bank of Cancer Autoimmunity and Epidemiology Research Laboratory at UTEP (University of Texas at El Paso), which were originally provided by our collaborator, Dr X.-X. Peng at Sun Yat-sen University, Guangzhou, China. This study was approved by the Institutional Review Board of UTEP and collaborating institutions.

All HCC patients were diagnosed according to the criteria described in a previous study (34), and had not received treatment with any chemotherapy or radiotherapy. Patients with CH and LC were followed up at least 18 months after collecting blood to exclude individuals with primary biliary cirrhosis and asymptomatic or clinically undetectable HCC. NHS were assembled from individuals at the same locality during annual health examinations, who had no obvious evidence of malignancy. In all 76 HCC patients, general information for 69 patients was available. Of the 69 HCC patients, 50 (72.5%) were male, and 19 (27.5%) were female. Mean age was 58.1±13.1 years (range, 24–78 years). Of these 69 patients, 51 (73.9%) were positive for HBV, 7 (10.1%) for HCV, and 4 (5.8%) for both HBV and HCV; 44 (63.8%) patients had a previous history of CH, 13 (18.8%) of LC, and 24 (34.8%) patients had no previous history of either CH or LC. Based on the Chinese guideline for liver cancer, 22 (31.9%) patients were in clinical stage I, 13 (18.8%) patients in stage II, 21 (30.4%) in stage III, 8 (11.6%) in stage IV, respectively and for 5 (7.2%) patients there was no available data on clinical stages. Of the 76 HCC sera, 63 had been tested by α-fetoprotein (AFP) in a previous study.

### Recombinant proteins and antibodies used in this study

The full-length recombinant protein GRP78 was commercially purchased (Abcam, Cambridge, MA). Polyclonal anti-GRP78 rabbit antibody and monoclonal anti-β-actin mouse antibody were purchased (Cell Signaling Technology, Inc., Danvers, MA). Horseradish peroxidase (HRP)-conjugated goat anti-human IgG, HRP-conjugated goat anti-rabbit IgG, HRP-conjugated goat anti-mouse IgG and FITC-conjugated goat anti-human IgG were purchased (Santa Cruz Biotechnology, Inc., Santa Cruz, CA). Anti-rabbit IgG Fab2 (Alexa Fluor 488) was purchased (Life Technologies, Grand Island, NY).

### Cell lines and cell extracts

Nine different tumor cell lines, human epidermoid carcinoma (Hep2), human hepatocellular carcinoma (HepG2), human hepatocellular carcinoma (SUN449), human breast cancer (SKBR3), human ovarian carcinoma (SKOV3), human lung epithelial adenocarcinoma (A549), human urinary bladder carcinoma (T24), human acute lymphoblastic leukemia (MOLT-4) and leukemia (KOPN63), were obtained from the Tumor Cell bank of our Laboratory and cultured following the specific protocol for each cell line. Cells grown in monolayers were solubilized directly in Laemmli’s sample buffer containing protease inhibitors. Solubilized lysates were briefly sonicated before electrophoresis on SDS-polyacrylamide gels.

### Enzyme-linked immunosorbent assay (ELISA)

Standard protocol for ELISA was used as described in our previous study (12). In brief, a 96-well microtiter plate (Thermo Scientific, Waltham, MA) was coated overnight at 4°C with recombinant GRP78 protein at a final concentration of 0.5 *μ*g/ml in phosphate-buffered saline (PBS). The antigen-coated wells were blocked with gelatin post-coating solution at room temperature for 2 h. Human sera diluted at 1:100 with serum diluent were incubated for 2 h at room temperature in the antigen-coated wells, followed by HRP-conjugated goat anti-human IgG. The substrate 2,2′-azino-bis-3-ethylbenzothiazoline-6-sulfonic acid (ABTS, Sigma-Aldrich, St. Louis, MO) was used as detecting reagent. The average optical density (OD) value at a wavelength of 405 nm was applied as data analysis. The cutoff value designating positive reaction was the mean OD of 86 NHS + 3SD.

### Western blotting

Denatured recombinant GRP78 protein and cancer cell lysates were electrophoresed on 10% SDS-PAGE and transferred to nitrocellulose papers, respectively. After blocking in PBS with 5% nonfat milk and 0.05% Tween-20 for 1 h at room temperature, the nitrocellulose papers were incubated overnight at 4°C with 1:200 dilution of human sera, 1:1000 dilution of polyclonal anti-GRP78 antibody and 1:500 dilution of monoclonal anti-β-actin mouse antibody, separately. HRP-conjugated goat anti-human IgG, HRP-conjugated goat anti-rabbit IgG and HRP-conjugated goat anti-mouse IgG were applied as secondary antibody at a 1:3000 dilution. The ECL-kit was used to detect immunoreactive bands according to the manufacturer’s instructions (Thermo Scientific, Waltham, MA).

### Indirect immunofluorescence assay (IIFA)

HEP-2 Antigen Substrate for IIFA Test System was incubated with dilution of sera (1:40) and preabsorbed sera for 30 min at room temperature or anti-GRP78 antibody (1:50) overnight at 4°C, separately. FITC-conjugated goat anti-human IgG or anti-rabbit IgG Fab2 (Alexa Fluor 488) was separately used as secondary antibody at a 1:100 dilution. Fluorescence microscope (Leica DM1000, Germany) was used for examination.

### Absorption of antibodies with recombinant protein

The diluted human sera (1:40) were incubated with recombinant protein (final concentration of recombinant protein in the diluted human sera was 0.01 *μ*g/*μ*l) overnight at 4°C, then centrifuged at 10,000 × g for 10 min. The supernatant was used for immunofluorescence assay.

### Immunohistochemistry (IHC) with tissue array slides

Liver cancer tissue array slide with normal tissue controls (12 cases/24 cores, including clinical stages and pathology grades) were purchased (US Biomax, Inc., Rockville, MD), and used to detect the expression of the GRP78 protein. Tissue array slides were deparaffinized with xylene and dehydrated with ethanol. Antigen retrieval was performed by microwave-heating methods in Trilogy™ pretreatment solution for 20 min. Avidin/biotin blocking solution were used to prevent nonspecific binding of antibodies. The sections were incubated with polyclonal anti-GRP78 antibody (1:50 dilution) for 1 h at room temperature. HRP Detection System (HRP streptavidin label and polyvalent biotinylated link) and DAB Substrate Kit were used as detecting reagents. After counterstaining with hematoxylin, the sections were dehydrated and mounted. The slides were observed by light microscopy.

### Statistical analysis

The mean OD value of each group of patients’ sera was compared using the Mann-Whitney U test; the frequency of autoantibody to TAAs in each group of patients’ sera was compared using the χ^2^ test with Fisher’s exact test, and two significant levels (0.05 and 0.01) were used. Methods for calculating sensitivity and specificity were used as previously described (35).

## Results

### Frequency and titer of autoantibodies against GRP78 in HCC

**Figure 1:**
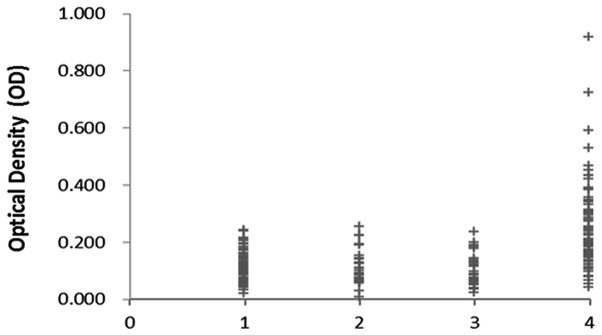
Titer of autoantibody against GRP78 in human sera by ELISA. The range of antibody titers to GRP78 was expressed as optical density (OD) obtained from ELISA. The mean + 3SD of NHS are shown in relationship to all serum samples. The mean OD value of NHS, CH, LC, HCC was 0.112, 0.111, 0.100, 0.247, respectively. Titer of anti-GRP78 in HCC is much higher than that in other types of sera (P<0.01). Lane 1, NHS; lane 2, CH; lane 3, LC; lane 4, HCC.

**Figure 2:**
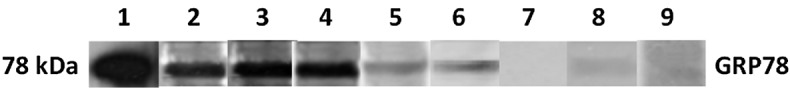
Western blot analysis with representative sera in ELISA. Lane 1, the polyclonal anti-GPR78 antibody was used as positive control. Lanes 2–4, three representative HCC sera which were positive in ELISA also had strong reactivity with GRP78 recombinant protein in western blot analysis. Lanes 5–6, two random LC sera had weak reactivity with GRP78 recombinant protein. Lanes 7–8, two random CH sera had weak reactivity and negative reactivity with GRP78 recombinant protein, respectively. Lanes 9, one random NHS had negative reactivity with GRP78 recombinant protein.

The full-length recombinant GRP78 protein was used as coating antigen in ELISA to screen autoantibodies against GRP78 in sera from patients with HCC, LC and CH, as well as NHS. In total, 76 sera from patients with HCC, 30 from LC, 30 from CH, and 86 sera from normal human individuals were used in this study. As shown in Table. I, the prevalence of autoantibody against GRP78 was 35.5% (27/76) in HCC, which was significantly higher than that in LC, CH, and NHS (P<0.01). Titer of anti-GRP78 antibodies in human sera are shown in Fig. 1. The titer of anti-GRP78 antibodies in sera from some of the HCC patients was much higher than that in other groups. The average titer of autoantibody against GRP78 in HCC sera was higher than that in HC, CH, and NHS (P<0.01). The ELISA results were also confirmed by western blot analysis. Fig. 2 shows that representative HCC sera with positive reaction to GRP78 in ELISA also have strong reactivity in western blotting compared to LC, CH, and normal sera, though weakly reactive bands were shown in some LC and CH sera. Of the 76 HCC sera, 63 were tested with α-fetoprotein (AFP). In 63 HCC sera tested with both anti-GRP78 autoantibody and AFP, 37 (58.7%) had an AFP level >100 ng/ml, 31 (49.2%) had an AFP level >200 ng/ml and 25 (39.7%) were positive with anti-GRP78 autoantibody. When both anti-GRP78 autoantibody and AFP (either >100 ng/ml or >200 ng/ml) were simultaneously used as diagnostic markers, 45 (71.4%, AFP >100 ng/ml) and 43 of 63 HCC sera (68.3%, AFP >200 ng/ml) were positive, respectively.

### Perinuclear intense staining pattern showing in HEP-2 cells by indirect immunofluorescence assay with representative positive HCC sera

**Figure 3:**
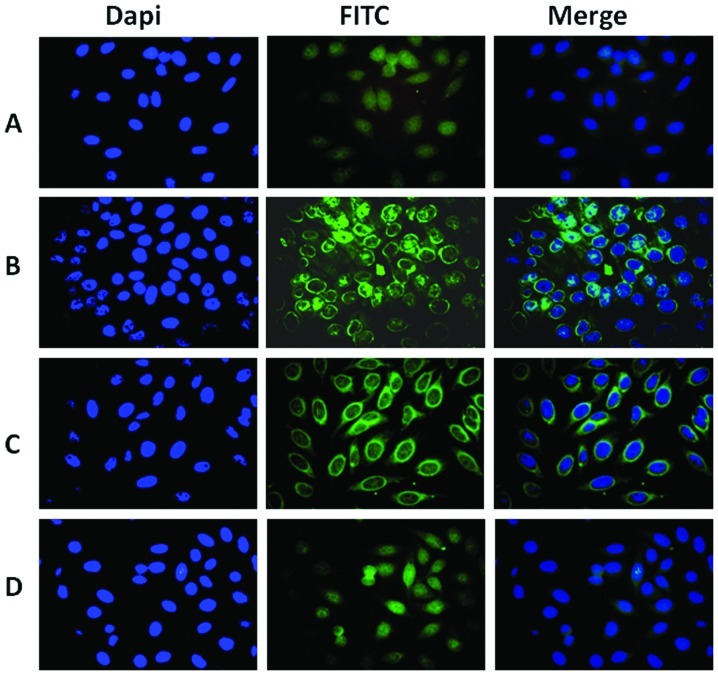
Representative immunofluorescence staining pattern of anti-GRP78 antibody positive HCC serum. (A) NHS were used as negative control; (B) Polyclonal anti-GRP78 antibody which showed a perinuclear immunofluorescence staining pattern was used as positive control; (C) A representative anti-GRP78 antibody positive HCC serum demonstrated an intense perinuclear staining pattern; (D) The same HCC serum used in panel C was pre-absorbed with recombinant GRP78. The fluorescent staining was significantly reduced.

To further confirm the reactivity of autoantibody in HCC sera to GRP78 and the intracellular location of GRP78, commercially purchased HEP-2 cell slides were used in indirect immunofluorescence assay to detect HCC sera with anti-GRP78 positive in ELISA. As shown in Fig. 3, a representative anti-GRP78 positive HCC serum had an intense perinuclear staining pattern, which was similar in pattern and location to that shown by polyclonal anti-GRP78 antibody. The fluorescent staining was significantly reduced when the same HCC serum pre-absorbed with recombinant GRP78 protein.

### Expression of GRP78 in liver cancer tissues and normal hepatic tissues by immunohistochemistry

**Figure 4:**
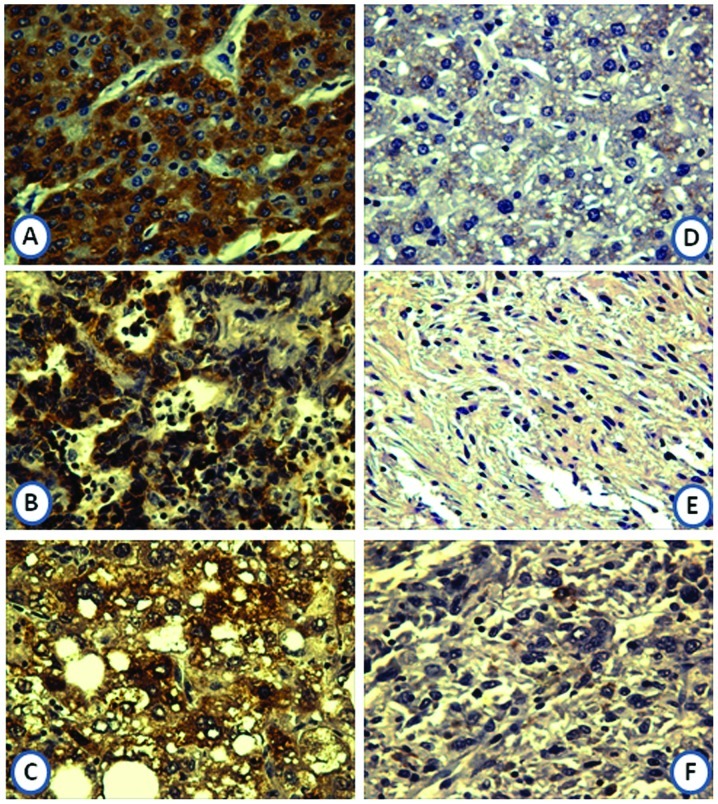
Expression of GRP78 in liver cancer and normal hepatic tissues by immunohistochemistry. The polyclonal anti-GPR78 antibody was used as primary antibody to detect the expression of GRP78 in liver cancer and normal hepatic tissues. (A) HCC tissue had strong positive staining; (B) a cholangiocellular carcinoma tissue was positively stained; (C) the clear cell carcinoma tissue was also positively stained; (D) a normal hepatic tissue had negative staining; (E) the malignant fibrohistiocytoma tissue had negative staining; (F) the angiosarcoma tissue was also negatively stained.

In the current study, the expression profile of GRP78 in liver cancer tissues and normal liver tissues was examined by immunohistochemistry with tissue array slides. Tissue array slides were commercially available for this study, including 5 HCC tissue specimens, 2 cholangiocellular carcinoma tissue specimens, 1 clear cell carcinoma tissue specimen, 1 malignant fibrohistiocytoma tissue specimen, 1 angiosarcoma tissue specimen and 2 normal hepatic tissue specimens. The polyclonal anti-GPR78 antibody was used as primary antibody to detect the expression of GRP78 in liver cancer and normal hepatic tissues. As a result, 3 of the 5 HCC tissues were positively stained (among these 3 positively stained tissues, 1 was strong positively stained, 1 was positively stained, and 1 was weak positively stained, respectively); 1 of the 2 cholangiocellular carcinoma tissues was positively stained; the clear cell carcinoma tissue was positively stained; 2 normal hepatic tissues were negatively stained; the malignant fibrohistiocytoma tissue and the angiosarcoma tissue were both negatively stained. Due to the small sample size of tissues in this study, it is difficult to establish a statistical analysis. The expression of GRP78 in liver cancer and normal hepatic tissues are shown in Fig. 4.

### Overexpression of GRP78 in different cancer cell lines

**Figure 5:**
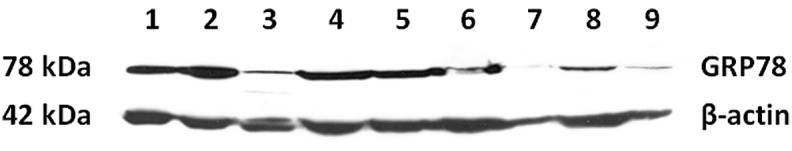
Nine types of tumor cell lines were analyzed by western blotting. The polyclonal anti-GPR78 antibody was used as probe. Hep2, HepG2, SKBR3, SKOV3 cell lines had strong reactivity, and SUN449, A549, T24, MOLT-4, KOPN63 cell lines had weak reactivity compared to cell lines which have clear reactive bands. 1, Hep2; 2, HepG2; 3, SUN449; 4, SKBR3; 5, SKOV3; 6, A549; 7, T24; 8, MOLT-4; 9, KOPN63.

To confirm the expression of GRP78 in different tumors, 9 tumor cell lines (Hep2, HepG2, SUN449, SKBR3, SKOV3, A549, T24, MOLT-4, KOPN63) were analyzed by western blotting. The polyclonal anti-GPR78 antibody was used as probe for this study. As shown in Fig. 5, Hep2, HepG2, SKBR3, SKOV3 cell lines had strong reactivity, and SUN449, A549, T24, MOLT-4, KOPN63 cell lines had weak reactivity compared to cell lines which have clear reactive bands.

## Discussion

GRP78 is well recognized as a vital ER molecular chaperone and regulator in the ER stress signaling pathway (13–15). Under the conditions or factors of ER stress-induction, the perturbation of ER homeostasis leads to the accumulation of misfolded proteins, which triggers the unfolded protein response (UPR) for cell survival (36). The 3 trans-membrane ER stress sensors PKR-like ER kinase (PERK), activating transcription factor (ATF) 6 and inositol requiring (IRE) 1 are dissociated from molecular chaperone GRP78, which interact with GRP78 in non-stressed cells in inactive forms, to activate the 3 distinct UPR signal pathways (15,36). These signaling pathways can reduce protein synthesis, upregulate the transcription of chaperone genes to ameliorate ER load and increase capacity of protein folding (37). GRP78 gene expression can upregulate similarly in rapidly growing tumors, which attributes to the tumor microenvironment of ER stress, such as glucose starvation, acidosis and hypoxia (13,37). Overexpression of GRP78 in a variety of cancer tissues compared with normal tissue has been shown in many studies (16–20). This study also revealed a high level of expression of GRP78 in the Hep2, HepG2, SKBR3, SKOV3 cancer cell lines and a relatively weak expression in the SUN449, A549, T24, MOLT-4, KOPN63 cell lines. Our study suggested that overexpression of GRP78 was not only in certain solid tumors, but also in leukemia (MOLT-4, KOPN63) cells, which further reveals the higher relevance of GRP78 with malignancy. In tumor cells, expression of GRP78 may play a critical role in proliferation, survival, anti-apoptosis and chemoresistance (18,21–27). Interfering PI3K/Akt signaling was shown as a major mechanism of GRP78 facilitating tumor growth and resisting apoptosis (38). Recent studies have also shown a significant correlation between GRP78 and poor cancer prognosis (18,20,22,23,39).

The expression profile of GRP78 in liver cancer and normal liver tissues was examined by immunohistochemistry in the current study. Overexpression of GRP78 in HCC tissues compared with normal tissues is shown in this study, which is consistent with the findings of previous reports (20,23,39). Expression of GRP78 in cholangiocellular carcinoma and clear cell carcinoma tissues has also been shown, but not in malignant fibrohistiocytoma and angiosarcoma tissues. It is difficult to establish a statistical analysis due to the small sample size of tissues in this study. Upregulation of GRP78 hinting at a poor prognostic outcome in HCC by promoting invasion and associating with venous infiltration, vascular invasion and intrahepatic metastasis has been reported (20,23,39). However, there is little information about autoantibodies against GRP78 in sera from HCC patients and a correlation between anti-GRP78 autoantibodies and HCC, although high levels of autoantibodies against GRP78 have been detected in the sera from patients with prostate and gastric cancer (31–33). In the present study, 35.5% of HCC sera showed immune response to GRP78 recombinant protein, but not in LC, CH, and NHS. The mean titer of autoantibody against GRP78 in the sera of HCC was significantly higher than that in other cohorts. The high frequency of autoantibodies against GRP78 in HCC sera in one way suggested that it could be used as a potential serological marker in the immunodiagnosis of HCC. Notably, when both autoantibody against GRP78 and AFP are used as diagnostic markers simultaneously, sensitivity can reach 71.4%, which is much higher than that when using either anti-GRP78 or AFP as a marker. Taken together, our data indicate that anti-GRP78 autoantibody might be useful as a complementary marker in conjunction with AFP in HCC diagnosis.

An important feature of GRP78 is that it can express on the cancer cell surface, but not on normal cells (28–30). It reveals a profile of tumor cells different from normal cells. Cell surface GRP78 serves as a receptor in cell signaling transduction, survival and anticancer therapeutic targeting (30,38). For example, α2-macroglobulin (α2M*) combined with cell surface GRP78 can activate downstream cell survival signaling in 1-LN prostate cancer cells (40). The combination of Cripto with cell surface GRP78 can promote tumor growth by suppression of transforming growth factor-β (TGF-β) signaling (41). Autoantibody against a fragment of GRP78 in sera from patients with prostate cancer can induce cell proliferation, which recognizes the same site of GRP78 on the tumor cell surface as the co-receptor with α2M* (32). The same autoantibody against GRP78 increases tissue factor pro-coagulant activity by binding to cell surface GRP78 (42). An antibody against the N-terminus of GRP78 (N-20 antibody) can compete for the same binding site of GRP78 with Cripto to inhibit Cripto signaling (43). A commercial polyclonal antibody directed against the C-terminus of GRP78 can induce apoptosis in melanoma cells (A375) and prostate cancer cells (1-LN and DU145), which may lead to the upregulation of p53, inhibition of NF-κB1 and NF-κB2 activation, and suppression of Ras/MAPK and PI3K/Akt signaling (44–46). Furthermore, autoantibody against GRP78 was reported to correlate with aggressive tumor behavior (14,31). In view of the critical role of autoantibodies against GRP78 in tumor cell survival, the high levels of anti-GRP78 autoantibodies detected in HCC sera in this study implied the close correlation between anti-GRP78 autoantibodies and HCC. Further study is needed to investigate the role of anti-GRP78 autoantibody in HCC, especially the role of GRP78 on the tumor cell surface. High titer of autoantibodies against GRP78 in HCC sera may have potential value for HCC immunotherapy.

Although the mechanism of emerging autoantibodies remains unclear, many studies have demonstrated that autoantibody production is related to abnormal expression of autoantigen (47). Autoantibodies can be detected in the sera from patients with autoimmune diseases and various types of cancer as well as from some healthy individuals (9,47). In the present study, autoantibody against GRP78 was also detected in some sera of LC and CH, but the titer of anti-GRP78 autoantibody was much lower than it was in the sera of HCC. High titer of autoantibody against GRP78 in HCC sera may be in accordance with ectopic expression of GRP78, especially expression on the surface of tumor cells. We propose that the presence of high titer of autoantibody against GRP78 in sera from chronic liver diseases could be a signal of malignancy. Monitoring of the anti-GRP78 autoantibody titer in high risk individuals of HCC could be valuable for the early detection of malignancy, though further studies are required.

As described above, the findings in this study suggest that autoantibodies against GRP78 may play a vital role in tumor cell survival. This study provides further information on autoantibodies against GRP78 in the sera of HCC, LC, CH, and healthy individuals, suggesting that anti-GRP78 autoantibody may be a potential diagnostic marker for HCC, especially in conjunction with AFP. Whether the monitoring of the autoantibody titer in high risk individual of HCC is necessary, remains to be confirmed.

## Figures and Tables

**Table I:** Frequency of autoantibody against GRP78 in human sera by ELISA.

Type of sera	No. tested	Autoantibody to GRP78 (%)
HCC	76	27 (35.5)^**^
LC	30	0
CH	30	0
NHS	86	0

Cutoff value, mean + 3 SD of NHS; P-value relative to NHS, CH, LC,

** P<0.01. HCC, hepatocellular carcinoma; LC, liver cirrhosis; CH, chronic hepatitis; NHS, normal human sera.
